# Evaluation of an open access software for calculating glucose variability parameters of a continuous glucose monitoring system applied at pediatric intensive care unit

**DOI:** 10.1186/s12938-015-0035-3

**Published:** 2015-04-24

**Authors:** Gábor Marics, Zsófia Lendvai, Csaba Lódi, Levente Koncz, Dávid Zakariás, György Schuster, Borbála Mikos, Csaba Hermann, Attila J Szabó, Péter Tóth-Heyn

**Affiliations:** First Department of Pediatrics, Semmelweis University, Bókay u. 53-54, Budapest, 1083 Hungary; MRE Bethesda Children’s Hospital, Bethesda u. 3, Budapest, 1146 Hungary; Department of Measurement and Automation, Kálmán Kandó Faculty of Electrical Engineering, Óbuda University, Bécsi út 96/B, Budapest, 1034 Hungary; Department of Anesthesia and Intensive Care, Semmelweis University, Kútvölgyi út 4, Budapest, 1125 Hungary; MTA-SE Pediatrics and Nephrology Research Group, Bókay u. 53, Budapest, 1083 Hungary

**Keywords:** Glucose variability, Glucose homeostasis, Continuous glucose monitoring, Critical care

## Abstract

**Background:**

Continuous Glucose Monitoring (CGM) has become an increasingly investigated tool, especially with regards to monitoring of diabetic and critical care patients. The continuous glucose data allows the calculation of several glucose variability parameters, however, without specific application the interpretation of the results is time-consuming, utilizing extreme efforts. Our aim was to create an open access software [Glycemic Variability Analyzer Program (GVAP)], readily available to calculate the most common parameters of the glucose variability and to test its usability.

**Methods:**

The GVAP was developed in MATLAB® 2010b environment. The calculated parameters were the following: average area above/below the target range (Avg. AUC-H/L); Percentage Spent Above/Below the Target Range (PATR/PBTR); Continuous Overall Net Glycemic Action (CONGA); Mean of Daily Differences (MODD); Mean Amplitude of Glycemic Excursions (MAGE). For verification purposes we selected 14 CGM curves of pediatric critical care patients. Medtronic® Guardian® Real-Time with Enlite® sensor was used. The reference values were obtained from Medtronic®^’^s own software for Avg. AUC-H/L and PATR/PBTR, from GlyCulator for MODD and CONGA, and using manual calculation for MAGE.

**Results:**

The Pearson and Spearman correlation coefficients were above 0.99 for all parameters. The initial execution took 30 minutes, for further analysis with the Windows® Standalone Application approximately 1 minute was needed.

**Conclusions:**

The GVAP is a reliable open access program for analyzing different glycemic variability parameters, hence it could be a useful tool for the study of glycemic control among critically ill patients.

**Electronic supplementary material:**

The online version of this article (doi:10.1186/s12938-015-0035-3) contains supplementary material, which is available to authorized users.

## Background

Continuous glucose monitoring (CGM) is primarily applied in diabetes care for both clinical investigations and decision making. There are several promising studies suggesting that CGM could be a useful method for monitoring of critically ill patients [1-5]. However, the current guideline does not recommend CGM-based therapeutic decisions under intensive care circumstances without surveillance. This guideline also advocates further investigations about the reliability of CGM devices in clinical settings [6]. The final goal of monitoring glucose changes in critically ill patients is to maintain blood glucose levels within a narrow range, i.e. avoiding fluctuations of glycemia. This goal could be achieved in many cases by insulin therapy, or even by cortisone supplementation. Intensive care glucose changes are resultant of multiple interactions of glucose load, insulin secretion, and stress conditions mediated by cortisone or catecholamine secretion or autonomic nervous system activity. There are several models to describe glucose-insulin interaction taking into account many factors such as insulin sensitivity, insulin clearance, endogenous glucose production etc. Common feature of all the insulin-glucose models from the minimal model [7] through the pharmacokinetic model [8] to the most complex Sorensen model [9] is the consideration of glucose concentrations in the interstitial compartment, being the interstitial fluid the field of cellular insulin action. In the process of developing an appropriate algorithm for glucose regulation in the intensive care unit, the variability of interstitial glucose changes could not be neglected.

Applying CGM devices in the clinical practice or research needs appropriate, goal oriented data handling methods. For instance Medtronic® CGM device can be evaluated by a Windows- or Web-based program (Medtronic® CareLink® Professional/Personal), which are widely used applications by physicians and diabetic patients. The main advantage of the above programs is that they provide clinically important graphical reports, trends, areas and different parameters of glucose homeostasis with individually adjustable threshold values. Reports contain, however, limited information as far as glycemic variability is concerned; most accepted parameters, such as Mean of Daily Differences (MODD), Mean Amplitude of Glycemic Excursions (MAGE) and Continuous Overall Net Glycemic Action (CONGA) are not available, raising an issue for researchers. Raw data can be retrieved from both Medtronic® CareLink® Professional and Personal, thus separate calculation of these parameters is possible. Recently, softwares have been developed and found useful for the calculation of glycemic variability. The GlyCulator is an application designed for the evaluation of glycemic variability based on data collected by means of a CGM device and the program has been made accessible in a web-based, interface independent version [10]. Unfortunately, in this tool glucose thresholds are not adjustable representing a disadvantage for research purposes. Another application called CGM-GUIDE^©^ (Continuous Glucose Monitoring-Graphical User Interface for Diabetes Evaluation) calculates the most extensively used glucose homeostasis parameters and variability metrics, exported from the CGM device in a standard Excel data format [11]. It provides a user-friendly graphical interface, but it is not widely available.

Recent studies suggested a linkage between glycemic variability and critical care mortality. Elevated MAGE and standard deviation (SD) were found to be associated with increased in-hospital mortality [12]. Signal et al. demonstrated that the odds of living had been higher for those patients who had spent more time in the normoglycemic range of 72–126 mg/dL [13]. On the basis of the above observations we carried out a clinical study at a pediatric intensive care unit (PICU) setting with the aim to investigate glycemic variability. For this purpose our team designed a CGM data handling and analyzer program called Glycemic Variability Analyzer Program (GVAP). In this study we evaluated the reliability of GVAP, compared to reference values and determined the applicability of the software based on its user documentation. In the near future we will make the software available for open access usage, with the advantages of open access source code, adjustable thresholds and graphical user interface.

## Methods

### CGM system

Interstitial glucose level was monitored by Guardian® REAL-Time (Medtronic®, USA) CGM. The flexible platinum Enlite® sensor was inserted in the subcutaneous tissue of the left or right lateral thigh and covered by transparent dressing. The calibration protocol can be found in our previous publication [14]. The study was approved by the Research Ethical Committee (number: TUKEB 2012/4) of the Semmelweis University, Budapest.

### Developmental model and software testing

Our applied software designed for special application has several different outputs and mathematical algorithms as for certain data source. For the purposes of the development, incremental method was applied and each algorithm was written and tested in decomposed way. Following the algorithm design the next step involved the programming environment selection. Our choice was influenced by the main task being rather mathematical, therefore special software package application seemed to be optimal. Accordingly, MATLAB® (MathWorks®, USA) software was chosen. The GVAP program was tested in three consecutive steps: during the (1) static test an expert programmer read the source code row by row; (2) dynamic test addressed the evaluated data and algorithm precision during process, throughout this step the source code was considered as a black-box; (3) verification.

### Algorithm

In this application the main input was data on glucose concentration. The data chart contained a sequential series of glucose concentrations at 5 minute intervals. Taking into consideration the occasionally missing values, (e.g. secondary to late calibration) a calculation of the missing data with linear interpolation was incorporated into the initial steps, when applicable. During the processing the following parameters were calculated based on their formulas (Table 1): Average area above/below the target range (Avg. AUC-H/L); Percentage spent Above/Below the Target Range (PATR/PBTR); CONGA; MODD; MAGE; Excursion Frequency (EF).

**Table 1:** **Definition of the calculated parameters of the GVAP**

**Name**	**Formula**	**Symbols**
Avg. AUC-H	$\frac{1}{T}{\displaystyle {\int}_0^T\left(G(t)-T{R}_H\right)dt,\mathrm{IF}\kern0.5em \mathrm{G}\left(\mathrm{t}\right)>\mathrm{T}{\mathrm{R}}_{\mathrm{H}}}$	G (t) - Glucose–time function
		Target Range-High - TR_H_
		Avg. AUC-H - Average exposure to hyperglycemia.
		Avg. AUC-H = 10 mg/dL means that during the observation, on average the CGM glucose exceeded TR_H_ limit by 10 mg/dL [23].
Avg. AUC-L	$\frac{1}{T}{\displaystyle {\int}_0^T\left(T{R}_L-G(t)\right)dt,\mathrm{IF}\kern0.5em \mathrm{G}\left(\mathrm{t}\right)<\mathrm{T}{\mathrm{R}}_{\mathrm{L}}}$	G (t) Glucose - time function
		Target Range-Low - TR_L_
MAGE +/−	$\sum \frac{\lambda }{n}\mathrm{IF}\kern0.5em \lambda >\mathrm{v}$	λ = amplitude of each glucose increase or decrease (nadir to peak / peak to nadir)
		n = number of observations
		ν = meaningful excursion (ME)
MAGE avg.		
EF		MAGE+/−: mean of the upward/downward excursions
		MAGE avg.: average of MAGEs
		EF sum of all excursions [15,24,16]
MODD	$\frac{{\displaystyle {\sum}_{t={t}_1}^{t_k}\left|B{G}_t-B{G}_{t-1440}\right|}}{k}$	BG: Blood Glucose
		k = number of observations where there is an observation at the same time 24 h (1,440 min) ago [25,26]
CONGA _(n)_	$\sqrt{\frac{{\sum^{t_k}}_{t=t1}{\left({D}_t-\overline{D}\right)}^2}{k-1}}$	k = number of observations where there is an observation n × 60 min ago
	where D_t_ = BG_t_-BG_t-m_ and	m = n × 60, in our program n = 1 [27,26]
	$$ \overline{D}=\frac{{\sum^{t_k}}_{t=t1}{D}_t}{k} $$	

**Figure 1:**
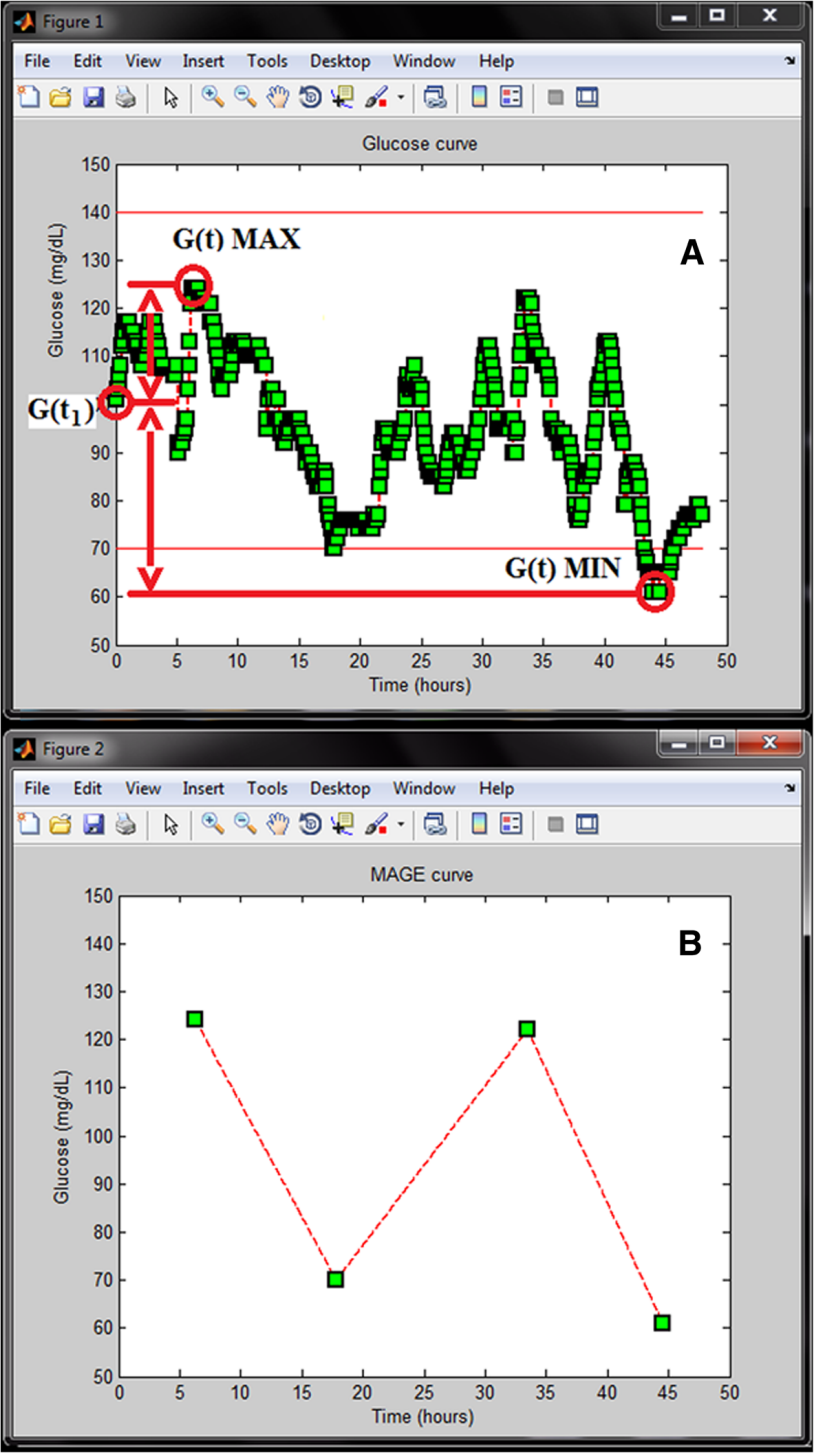
An example of a CGM curve with its MAGE curve. **A** represents a CGM curve. In some cases from the first glucose concentration (G (t_1_)) both the glucose maximum (G (t) MAX) and the minimum glucose values (G (t) MIN) did not exceed the meaningful excursion (ME = 45 mg/dL) [G (t) MAX - G (t_1_) and G (t_1_)-G (t) MIN ≤ ME], however, the entire curve contained MEs. On these occasions the program set the new starting point - in this case G (t) MAX. **B** shows the MAGE curve.

**Figure 2:**
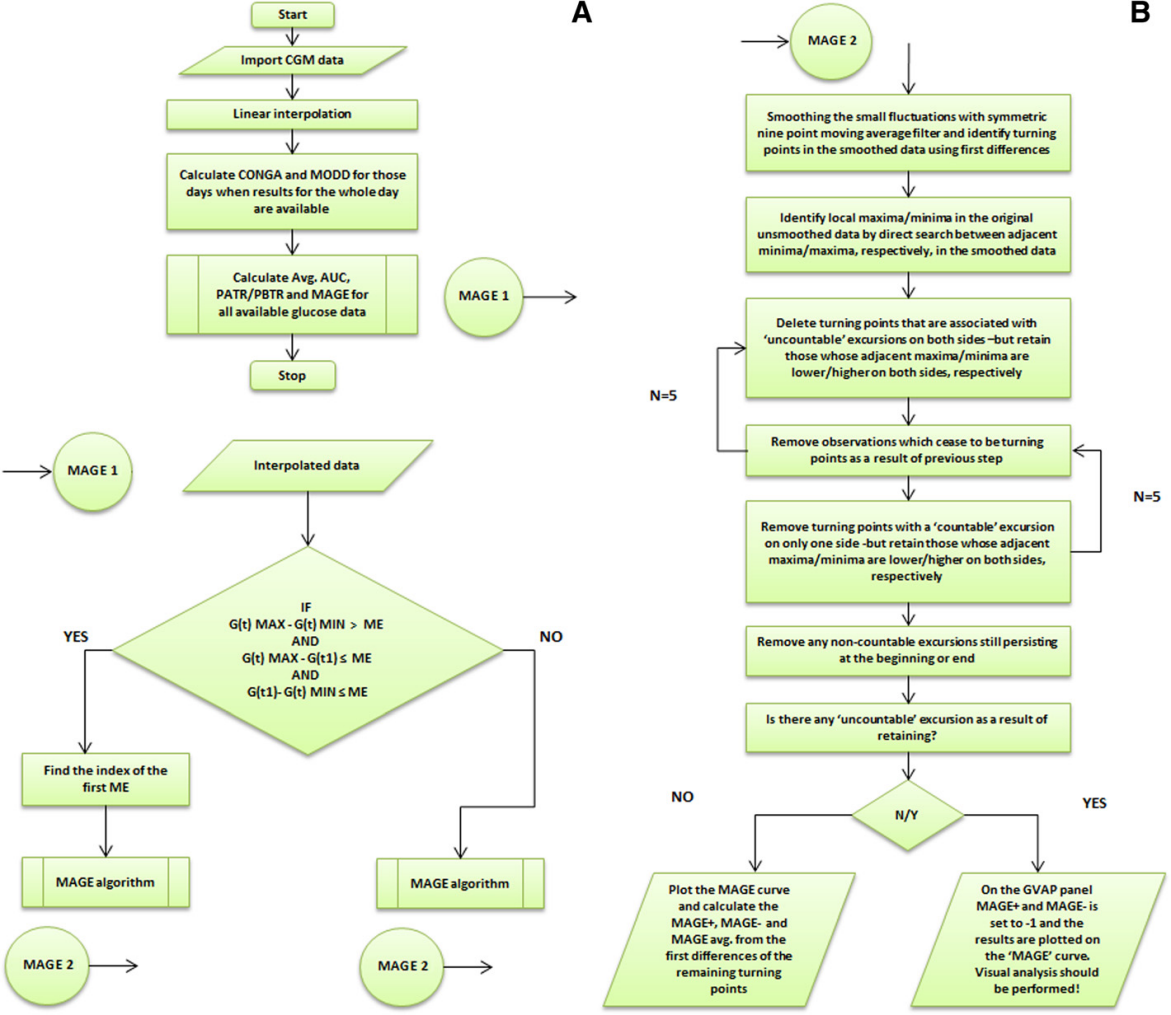
The algorithm. **A** and **B** show the entire algorithm of the Glycemic Variability Analyzer Program. The MAGE algorithm was evolved based on a previous report by Baghurst et al. [15].

**Figure 3:**
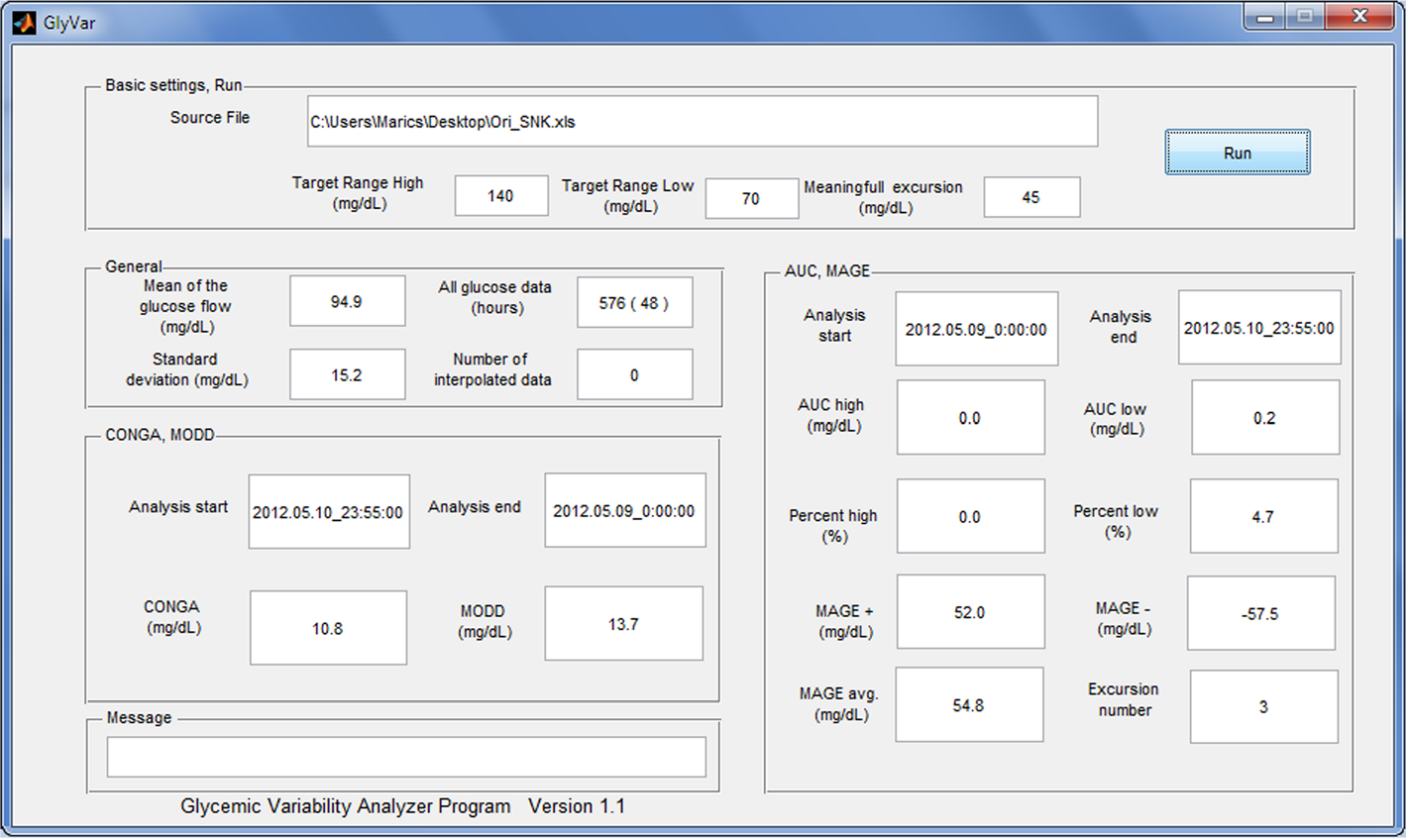
The control panel of the program. The control panel allows the users to set the required target range and meaningful excursions. The coherent results are in separate boxes. The user documentation provides more details.

Within the whole programming process, the formulation of MAGE was found to be the biggest challenge. Previously published algorithms were studied [15,16] and one of them (Baghurst’s algorithm, Approach 1) was opted for use with minor modifications. The MAGE algorithm embraced three main steps. The first element was the identification of the turning points of the glucose data. Then, the turning points that were associated with uncountable excursions on both sides were deleted, while those whom adjacent maxima/minima were lower/higher (‘W’ and ‘M’ pattern) on both sides, were retained. The third step comprised of the deletion of the turning points with countable excursions on only one side. While keeping the backbone of the Baghurst’s algorithm [15] two modifications were applied in our study design, as follows. In some cases, taking the first point of the glucose data as reference, the program could not identify a meaningful excursion (ME), although the data set itself contained MEs. For this context the program identified a new starting point. Moreover, when the program recognized the first ME, it rechecked the glucose curve whether within the starting point and the first identified ME any MEs were missed (Figure 1). The second modification affected the turning points. Our algorithm retained the “W” and “M” patterns throughout the calculation in contrary to the original method, which deleted them during the second step. As a result of retaining certain turning points, the theoretical situation could arise, when the final “MAGE” curve contained excursions below the threshold of ME. For this latter scenario the program was designed to alert the users as follows, “Visual analysis should be performed, see User Documentation”. It should be noted, though that this scenario occurred only in test circumstances. Figure 2 shows the entire algorithm and Figure 3 presents the control panel of the GVAP.

### Validation of the program

For validation of the program we used 14 CGM curves of clinical patients, and further 6 self-edited curves were evaluated for MAGE control with the aim to analyze extreme situations. CGM measurements were carried out at the PICU of the 1st Department of Pediatrics, Semmelweis University, Budapest, between 2011 February and 2013 December. Our study was approved by the local research ethical committee. The CGM data were collected by CareLink® Professional/Personal software in .xls file format. For the analysis 48-hour long periods were selected, where the Avg. AUC-H and/or the Avg. AUC-L was > 0. Missing data was not an exclusion criterion, data interpolation was permitted.

Glucose variability parameters calculated by our software were compared with the results of validated programs: 1. CareLink® Professional/Personal (Avg. AUC-H/L, PATR, PBTR), 2. GlyCulator (CONGA, MODD). For MAGE (MAGE avg.) calculation manual method was applied, as reference. Manual calculation followed the definition of MAGE by Peter Baghurst [15]. In our study instead of standard deviation (SD) of the sample we counted excursions exceeding 45 mg/dL in both directions. However, GVAP can calculate the SD of the glucose data as well, and the user can overwrite the default value of ME (45 mg/dL) when required for certain research purposes.

### Statistical analysis

The accuracy of the GVAP was evaluated by using correlation and Bland-Altman analysis, Pearson or Spearman correlation, as appropriate. Statistical analysis was carried out by SPSS® 13.0 software (SPSS® Inc., Chicago, IL, USA) and Microsoft® Excel 2010.

### User documentation

In addition to the source code we also provided a brief user documentation (Additional files 1 and 2), which had been tested by two independent users (DZ, CsH) without any knowledge of the GVAP or MATLAB®.

## Results and discussion

### CGM system

An average of 3.3 calibrations per 24 hours (range: 2–6) were performed in the selected 48-hour long periods. At the implantation site of the platinum sensor no undesirable events (bleeding, irritation, decubitus and infection) occurred.

### Verification of the program

**Figure 4:**
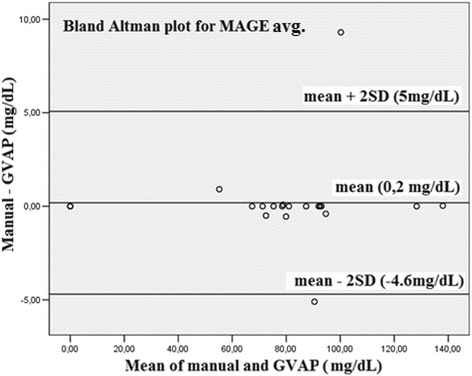
Bland-Altman plot. Average glucose concentration versus MAGE (Manual) – MAGE (GVAP) glucose difference.

Among the 14 patients 7 had missing data. The average amount of missing data per 24-hour recordings was 1.9 (range: 0–9), and glucose flow contained 286.1 (range: 279–288) points daily, on average. Correlation analysis between GVAP and validated programs was performed. Pearson analysis (MAGE avg.), Pearson analysis after logarithmic transformation (Avg. AUC-H, PATR) and Spearman analysis (Avg. AUC-L, PBTR) were applied. Correlation coefficient was above 0.99 for all measured parameters (Table 2). Compatibility of the methods was investigated by Bland-Altman analysis that found outliers (above 2 SD) for 5 variables (Table 3, Figure 4).

**Table 2:** **Validation results of the GVAP**

**Method**	**Parameter**	**N**	**GVAP (r values)**	**p level**
GlyCulator	CONGA	14	1	p < 0.001
	MODD	14	1	p < 0.001
Medtronic®	Avg. AUC-H	14	1	p < 0.001
	PATR	14	0.995	p < 0.001
	Avg. AUC-L	14	0.999	p < 0.001
	PBTR	14	0.992	p < 0.001
Manual	MAGE avg.	20	0.997	p < 0.001

**Table 3:** **Descriptive statistics on the accuracy of the GVAP**

		**Reference**				**Difference (Reference-GVAP )**				
	**N**	**Max.**	**Min.**	**Mean**	**SD**	**Max.**	**Min.**	**Mean**	**SD**	**Outliers**
CONGA (mg/dL)	14	43	9	20.4	11.6	0.7	-0.1	0.1	0.2	1
MODD (mg/dL)	14	90.7	11.3	29.5	20.7	1.7	-0.5	0.2	0.5	1
Avg. AUC-H (mg/dL)	14	27.5	0	3.4	7.6	0.4	0	0	0.1	1
PATR (%)	14	48.5	0	8.8	14.2	0	-1.6	-0.5	0.6	0
Avg. AUC-L (mg/dL)	14	3.3	0	1.3	1.8	0.1	0	0	0.1	0
PBTR (%)	14	24.5	0	14	15	1.9	-0.2	0.3	0.6	1
MAGE avg. (mg/dL)	20	128.3	0	78.9	33.1	9.3	-5.1	0.2	2.4	2

Difference: difference of the reference measurement and GVAP, Outliers: number of points out of 2 SD. Time of the observation was 48 hours.

### User documentation

Taking into account the feedback from the examiners the user documentation was adequately edited. Without any previous MATLAB experience the initial executions of the program lasted approximately 30 minutes. The duration of further examinations carried out with the Windows® Standalone Application was 1 minute, on average. The most frequent practical default was identified as typing mismatch, when selecting the excel-file to run the analysis.

### Clinical implementation

**Figure 5:**
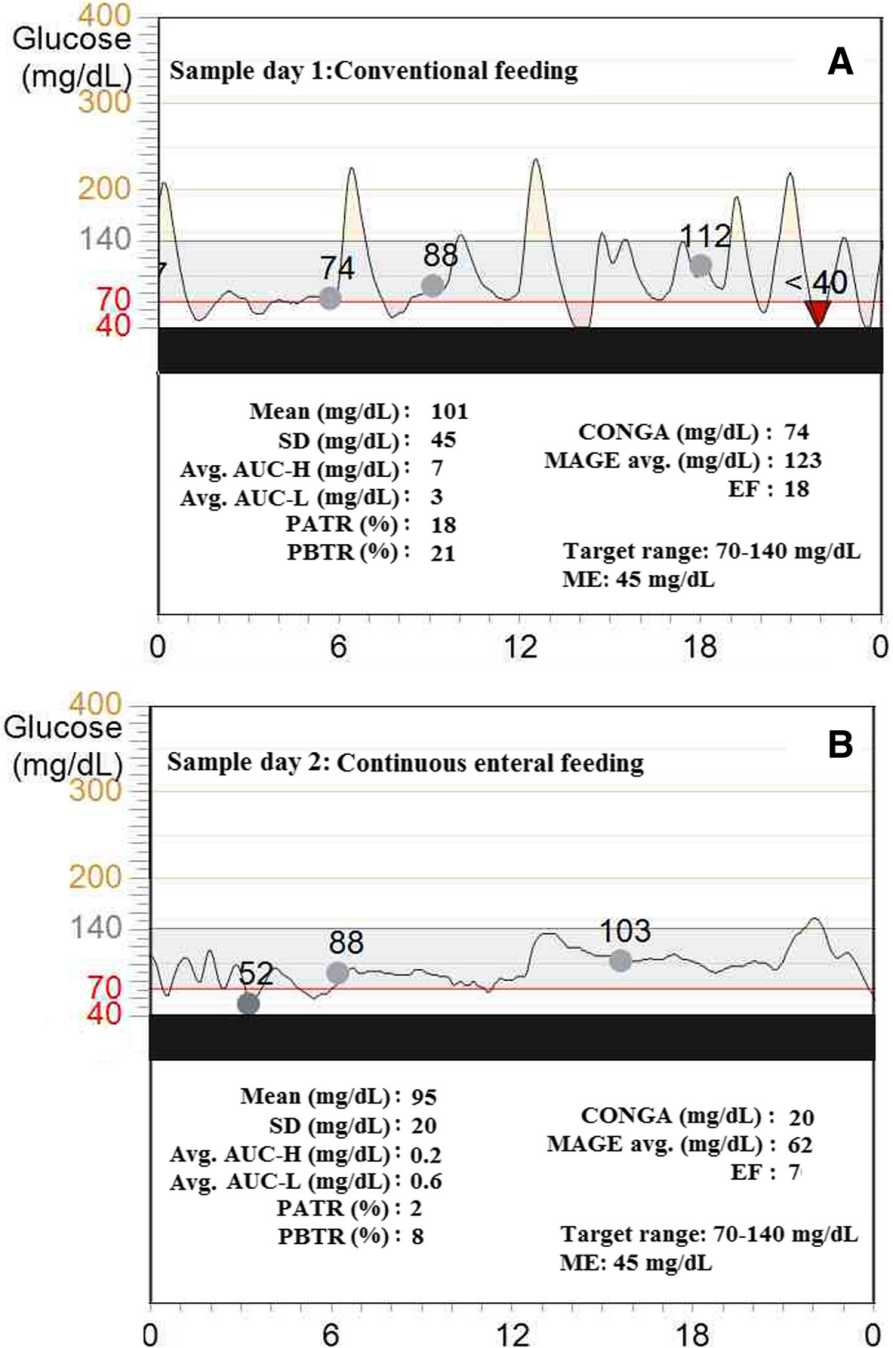
Sample days of a patient with dumping syndrome. **A**, during conventional feeding the patient presented severe hypoglycemic episodes. **B**, after introduction of continuous enteral feeding the glycemic variability parameters and severity of hypoglycemia reduced. The sample curves represent a 24-hours long period; the dots are the calibration points.

For clinical consideration we demonstrate two CGM curves from a patient with severe symptomatic hypoglycemia (glucose < 40 mg/dL) due to dumping syndrome. With the usage of conventional fractionated oral feeding, CGM revealed several hypoglycemic episodes. On the basis of CGM results, continuous feeding regime was introduced, resulting in significantly decreased variability metrics of the glucose curve (Figure 5). In the above case the CONGA seemed to be the most representative parameter that evaluated the glucose difference hour by hour, being sensitive for the short term variability. Its value decreased from 74 to 20 mg/dL after adjustment of treatment as rapid excursions were significantly reduced. Furthermore, in a group of 21 PICU patients we have found significant correlation of the mortality outcome index PRISM III and of MAGE avg. (r = 0,55; p < 0.05, unpublished observation by our group).

## Discussion

The last decade emerged an expanding interest on glucose homeostasis disorders in patients needing intensive care. Recently, glucose variability has been pointed out to play a significant role in intensive care morbidity and mortality [12,13]. However, there has been lack of evidence of which variability parameters could best characterize the severity of illness. The demand for more accurate clinical follow up (incorporating more frequent glucose sampling) has led to the introduction of several glucose variability parameters. The CONGA and MODD were usually derived from CGM measurement, while others such as Avg. AUC, MAGE, PATR/PBTR, SD and glycemic lability index (GLI) not. Initially for research purposes, then for clinical monitoring it is essential to create a complex tool of parameters that could be analyzed by a suitable program. Our aim in the present study was to unify the most commonly used parameters of glucose variability in a freely accessible application. Prior to further research practices or introduction to clinical use, we felt it necessary to describe the development process and publish the first pilot test results.

In 2001 Van den Berghe demonstrated that tight glycemic control (TGC) reduced mortality in critically ill cardiac surgical patients, however, subsequent investigations did not confirm this benefit consequently [17-19]. The major disadvantage of TGC is the more frequent occurrence of hypoglycemia, nevertheless, TGC, based on STAR-Liege or SPRINT protocol could diminish the prevalence of hypoglycemic episodes [20,21]. In the future the routine use of CGM with sophisticated glucose-insulin algorithms might contribute to safer implementation of TGC among critically ill pediatric and adult patients. However, further research is needed to identify more factors, which can potentially contribute to the elevated glucose variability. CONGA refers mainly to the within day variability, while MODD reflects on the interday variability. MAGE, Avg. AUC-H/L and PATR/PBTR provide general interpretation on the glycemic homeostasis. GVAP can give explanation to so far unresolved queries, e.g.: (1) how do the most common critical care diagnostic or therapeutic procedures [e.g. bronchoscopy, necrectomy in burned patients, epidural analgesia] affect the short term glucose variability (CONGA); (2) how do the perioperative fluid and feeding management influence the MODD, (3) what common pitfalls of the different intravenous insulin protocols can be justified (MAGE, Avg. AUC-H/L, PATR, PBTR).

On comparison of our program with two existing programs for glucose variability analysis we have found a good correlation between the results of all tested parameters. Literature data varies slightly with regards to MAGE definitions and programming of MAGE calculation. Therefore, we applied manual calculation based on Baghurst’s definition and not compared GVAP with the MAGE calculations of GlyCulator.

Agreement between the reference programs and GVAP was investigated with Pearson, Spearman correlation and Bland-Altman analysis. We have found strong correlation, but outliers were identified in 5 variables. In the MAGE avg. group we observed two considerable alterations (9,3 and 5,1 mg/dL) between GVAP and manual calculation, as reference, due to error of manual calculation (Figure 4). It seemed that the analysis of high variability recordings were more reliable and simple with GVAP, compared to the traditional manual way. As for the other variables outliers were not clinically significant despite of the statistical difference. Possible explanations for the differences were data loss at unit conversion (mmol/L to mg/dL), missing values, or linear interpolation.

It should not be ignored that both the interpolation and the calibration may have significant effect on glucose variability. In case of missing data the linear interpolation can falsely decrease the variability as a result of the filling process of the unknown points. The calibration is the other crucial point. For example, if there is a 50 mg/dL difference between the reference and the subcutaneous glucose concentration, the CGM processor evaluates the calibration value and the parallel subcutaneous glucose concentration, then corrects the actual subcutaneous concentration. In this theoretical case it may cause a sudden step in the subcutaneous glucose curve, resulting in falsely elevated glucose variability. These phenomenon unfortunately accompany CGM measurements, however, their frequency could be reduced by more frequent calibration [22]. In view of the above, in clinical trials the researchers should calculate the general accuracy of the CGM with Pearson correlation, Clarke’s error grid and Bland-Altman analysis. Moreover, the number of interpolated data should also be added. GVAP gives the total number of glucose concentrations and the numbers of interpolated data (Figure 3). In one section up to 21 glucose values can be interpolated.

Several advantages of the program designed by us can be highlighted. The adjustable threshold can be useful in the investigation of various clinical questions; on the other hand the graphical representation of the glucose curves can help the interpretation of the variability parameters. Minor limitations can also be spotted: GLI was not taken into account during the developing process, GVAP does not give any information about the accuracy of the CGM measurement, and the installation of MATLAB software is required for running of GVAP.

## Conclusions

Our program provides a user-friendly option for researchers who require detailed analysis of the continuous glucose monitoring glucose curve. In the future, this application may help to present more detailed information on glucose homeostasis disorders of patients in the intensive care setting.

## Additional files

Additional file 1:
**Contains the full source code of the GVAP (GlyVar_Script.zip -** http://sourceforge.net/projects/glyvariab/files/?source=navbar **).**
Additional file 2:
**Contains the user documentation (User documentation for GVAP.doc -** http://sourceforge.net/projects/glyvariab/files/?source=navbar **).**

## Abbreviations

Avg. AUC-H: Average area above the target range
Avg. AUC-L: Average area below the target range
avg.: Average
CGM: Continuous Glucose Monitoring
CGM-GUIDE: Continuous Glucose Monitoring-Graphical User Interface for Diabetes Evaluation
CONGA: Continuous Overall Net Glycemic Action
EF: Excursion Frequency
GLI: Glycemic Lability Index
GVAP: Glycaemic Variability Analyser Program
MAGE: Mean Amplitude of Glycemic Excursions
ME: Meaningful Excursion
MODD: Mean of Daily Differences
PICU: Pediatric Intensive Care Unit
PATR: Percentage Spent Above the Target Range
PBTR: Percentage Spent Below the Target Range
SD: Standard Deviation
TGC: Tight Glycemic Control
