# Supplementary figures and images for: Evaluation of an open access software for calculating glucose variability parameters of a continuous glucose monitoring system applied at pediatric intensive care unit
Source: Biomed Eng Online. 2015 Apr 24;14:37. doi: 10.1186/s12938-015-0035-3 (PMC4416329; doi:10.1186/s12938-015-0035-3)

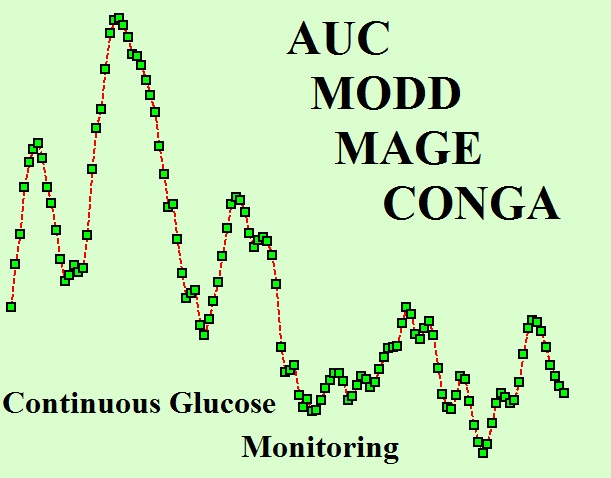

Supplement: Additional file 1: — Contains the full source code of the GVAP (GlyVar_Script.zip - http://sourceforge.net/projects/glyvariab/files/?source=navbar ). [file 12938_2015_35_MOESM1_ESM.zip › CGM Vaiab.jpg]
