# Supplementary material for: Evaluation of an open access software for calculating glucose variability parameters of a continuous glucose monitoring system applied at pediatric intensive care unit
Source: Biomed Eng Online. 2015 Apr 24;14:37. doi: 10.1186/s12938-015-0035-3 (PMC4416329; doi:10.1186/s12938-015-0035-3)
Supplement: Additional file 2: — Contains the user documentation (User documentation for GVAP.doc - http://sourceforge.net/projects/glyvariab/files/?source=navbar ). [file 12938_2015_35_MOESM2_ESM.doc]

**User documentation for the Glycemic Variability Analyzer Program**

Content

[Basic Information 2](#__RefHeading___Toc410053839)

[1. Specification 2](#__RefHeading___Toc410053840)

[2. Input format 2](#__RefHeading___Toc410053841)

[3. Method 3](#__RefHeading___Toc410053842)

[4. Perform Analysis 4](#__RefHeading___Toc410053843)

[5. Settings and Results 6](#__RefHeading___Toc410053844)

[6. Messages 7](#__RefHeading___Toc410053845)

[References 8](#__RefHeading___Toc410053846)

[Appendix: How to make Windows Standalone Application? 9](#__RefHeading___Toc410053847)

# Basic Information

The software/code is ***free for non-commercial use***. It was developed at the First Department of Pediatrics, Semmelweis University and revised at the Institute of Instrumentation and Automation, Kandó Kálmán Electrical Engineering Faculty, Óbuda University.

*Name and Version*: Glycemic Variability Analyzer Program 1.1

*Developmental environment*: MATLAB® 2010b

*Software requirement*: MATLAB® 2010b

*Developer*: **Gábor Marics** M.D. and Electrical Engineer (BSc)

*Email:* marics.gabor@gmail.com

*Last updated:* 2014 January

# Specification

The Glycemic Variability Analyzer Program calculates parameters associated with the glycemic variability. The following parameters can be determined (the definition of the parameters can be found in the literature ):

- Average Area below/above under curve (Avg. AUC-high/low, AUC-H/L).
- Percentage spent below the target range (PBTR, Percent low %).
- Percentage spent above the target range (PATR, Percent high, %)
- Continuous overall net glycemic action (CONGA)
- Mean of daily differences (MODD, mg/dL)
- Mean amplitude of glycemic excursions (MAGE+, MAGE-, MAGE avg., mg/dL)
- Excursion frequency (EF)

# Input format

For the correct analysis glucose flow must be provided in a specified excel table (.xls). It must contain four columns and at least 101 rows. The first row provides the labels from the left: Date, Time, Glu (Glucose - mg/dL), Index. The values are started from the second row. The Date and the Time should be in text format. The Index is started from 1 (Figure 1). The time interval must be 5 minutes within two consecutive rows. The glucose concentration must be within *30-500* mg/dL. The missing glucose data is calculated by *linear interpolation*, however the glucose flow should contain the first and the last glucose concentrations (Figure 2).


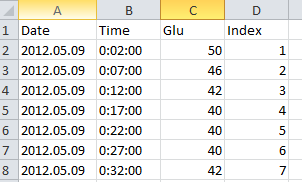


Figure 1. Input format.

# Method

The detailed algorithm of the program can be found in our research article (...). However one additional figure may be useful for the users. For the calculation of AUC-H/L, PATR/PBTR and MAGE the program uses all the available data, but for CONGA and MODD only result from days with measurements available for the whole day can be included (Figure 2).


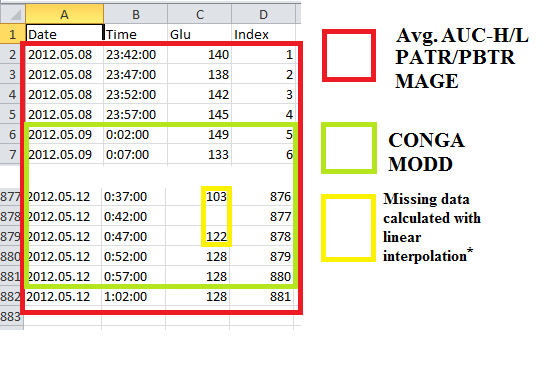


Figure 2. Time intervals for the different parameters.

# Perform Analysis

The MATLAB is a highly efficient programmable environment. With MATLAB one is able to run different programs and make windows standalone applications as well. Before the first application one should check weather *all necessary files are locted in the same folder*. ***Necessary files:*** GlyVar.m (main file); GlyVar.fig; FIUE.m; FSCD.m; IFS.m; MADD.m; MADOO.m; SNPF.m; LININCODE.m.

Without MATLAB experience at first one should launch the MATLAB. Please follow the instructions!

- Open the main MATLAB file (GlyVar.m).


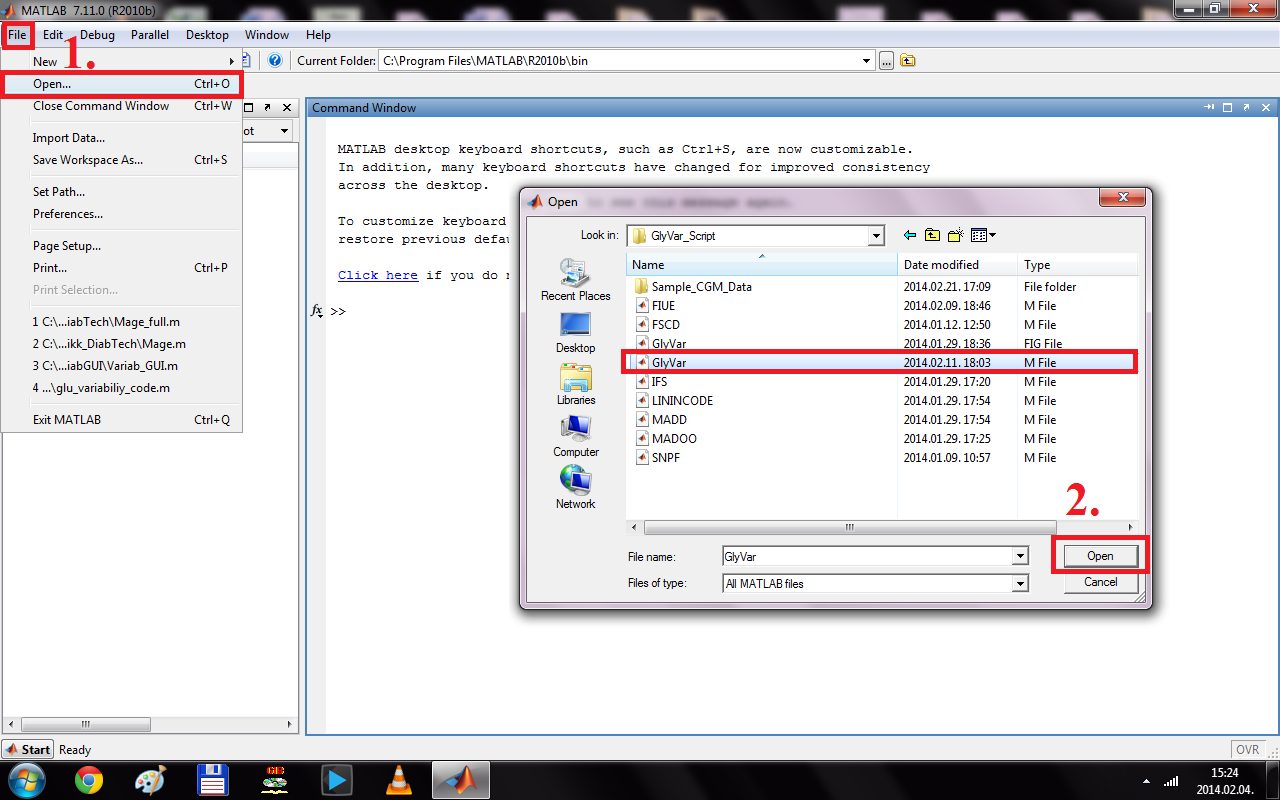


Figure 3. Home screen of MATLAB 2010b; opening process of the main file.

- Run the GlyVar.m file (main file).


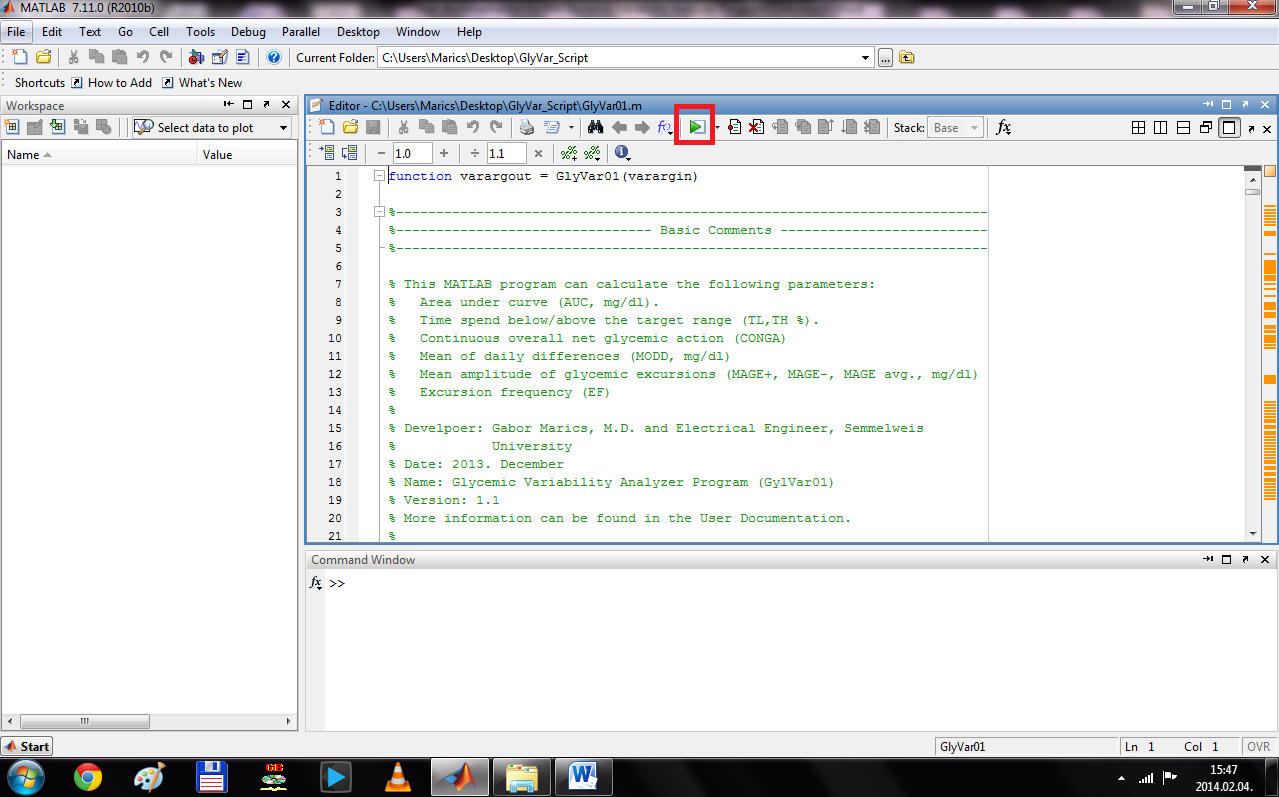


Figure 4. Running the GlyVar.

- Import the CGM data (eg. open Test1.xls file). This file can be found in the Sample _CGM_Data folder.


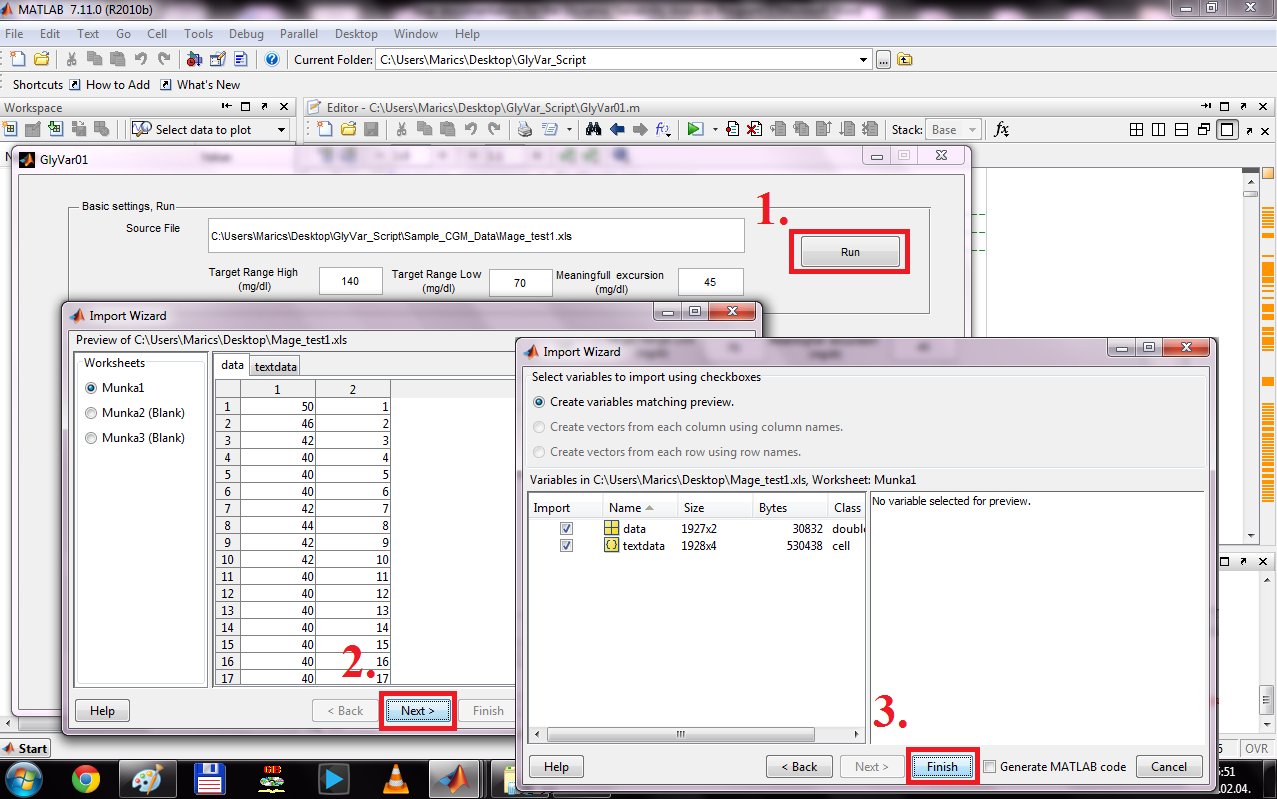


Figure 5. Data import.


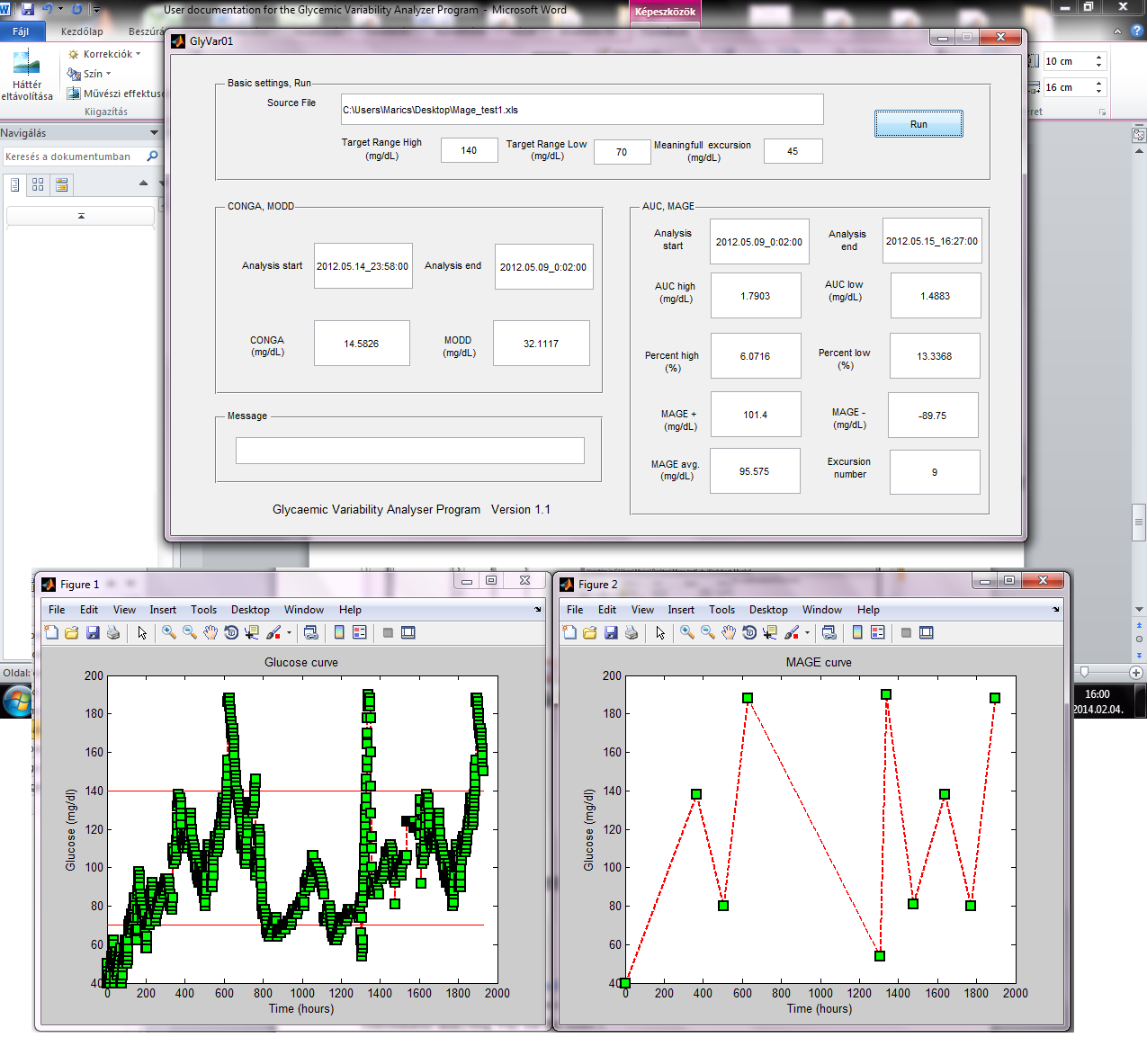


Figure 6. Final results.

# Settings and Results

Before the analysis parameters can be reset when needed:

- 50 mg/dl< ***Target Range*** < 240 mg/dL
- ***Meaningful excursion***, 18 mg/dl < ME< 200 mg/dL)

All results can be found in the control panel after the execution finished. If any parameter cannot be interpreted it is set to -1 (eg.: data containing results of a period less than a whole day therefore CONGA and MODD are set to -1).

Messages

Before the execution the program evaluates the input data and the settings (eg.: ME). In case of any problem an alert message appears on the control panel.

E.g.: If the ME value is above 200 mg/dL or less than 18 mg/dL

- Inputs: Meaningful excursion must be within 18-200 mg/dL.


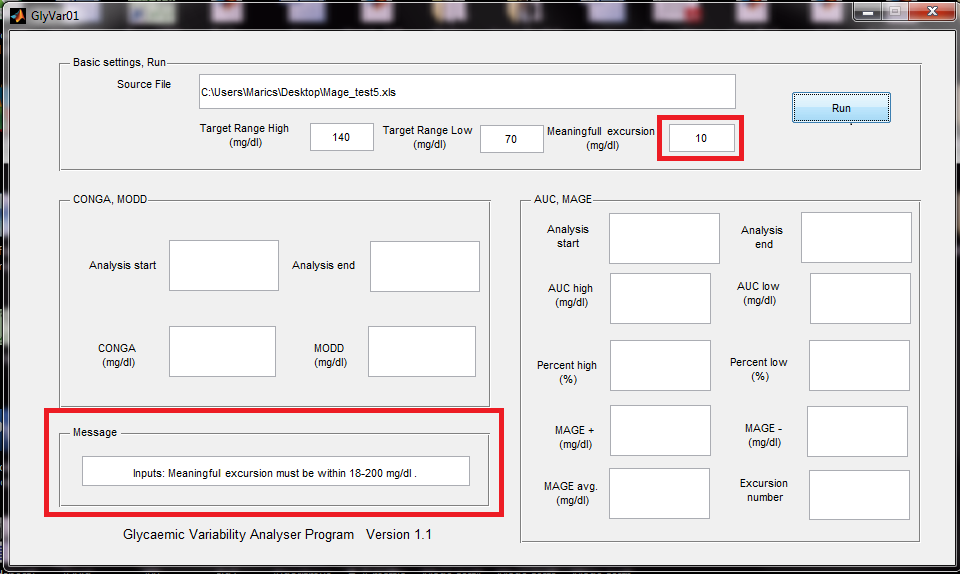


Figure 7. Message to the user.

***Further examples:***

If the source file is empty

- Inputs: There are no inputs

If the source file format is incorrect (see input format)

- Inputs: Data format is incorrect

If the source file contains less than 100 glucose data

- Inputs: Data must contain more than 100 elements

If the glucose flow contains values above 500 mg/dL or less than 30 mg/dL

- Inputs: glucose values must be within 30-500 mg/dL

If the Target Range High is above 240 mg/dL or Target Range Low is below 50 mg/dL

- Target range must be within 50-240 mg/dL

# References

1. Siegelaar SE, Holleman F, Hoekstra JB, DeVries JH: **Glucose variability; does it matter?***Endocrine reviews* 2010, **31**(2):171-182.

2. Baghurst PA: **Calculating the mean amplitude of glycemic excursion from continuous glucose monitoring data: an automated algorithm**. *Diabetes technology & therapeutics* 2011, **13**(3):296-302.

# Appendix: How to make Windows Standalone Application?

The MATLAB can make windows standalone application from the source code turning it to a user-friendly program.

- Windows application step by step:


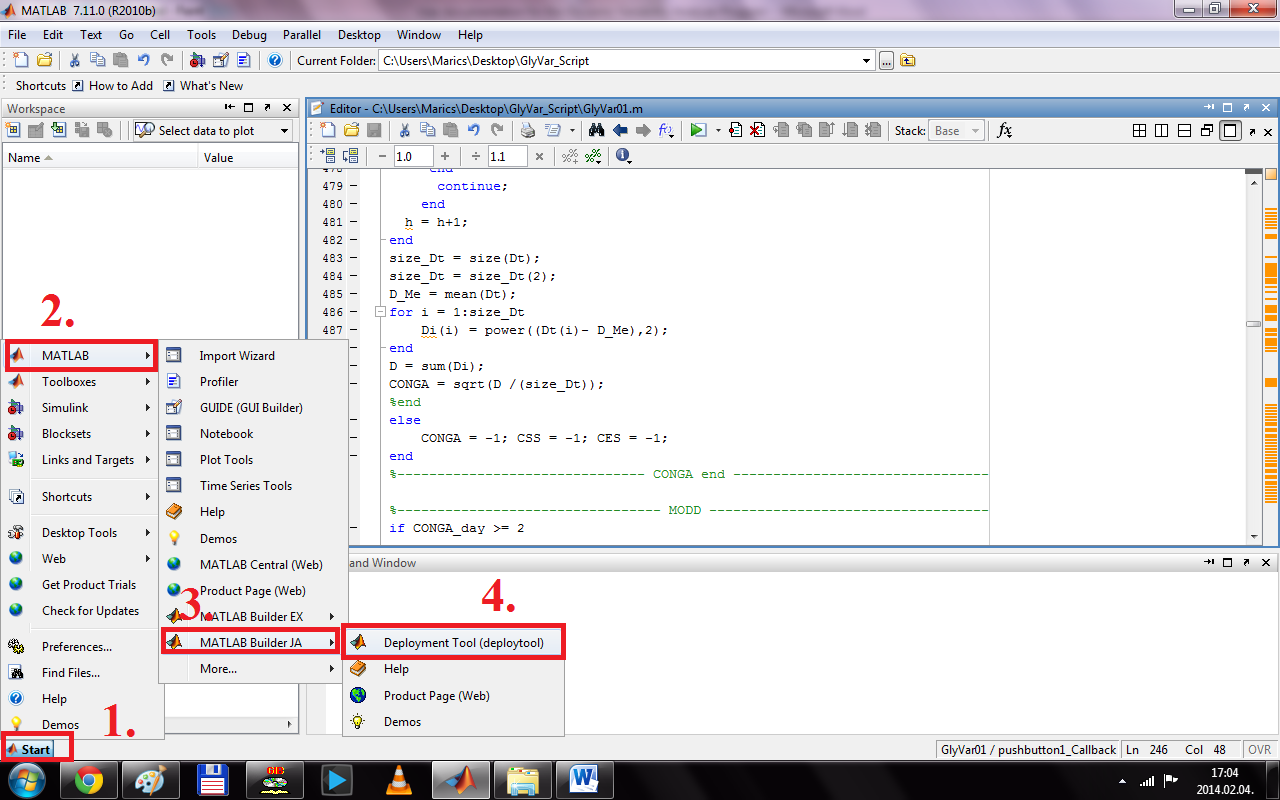


Step 1: Basic steps toward the project building.


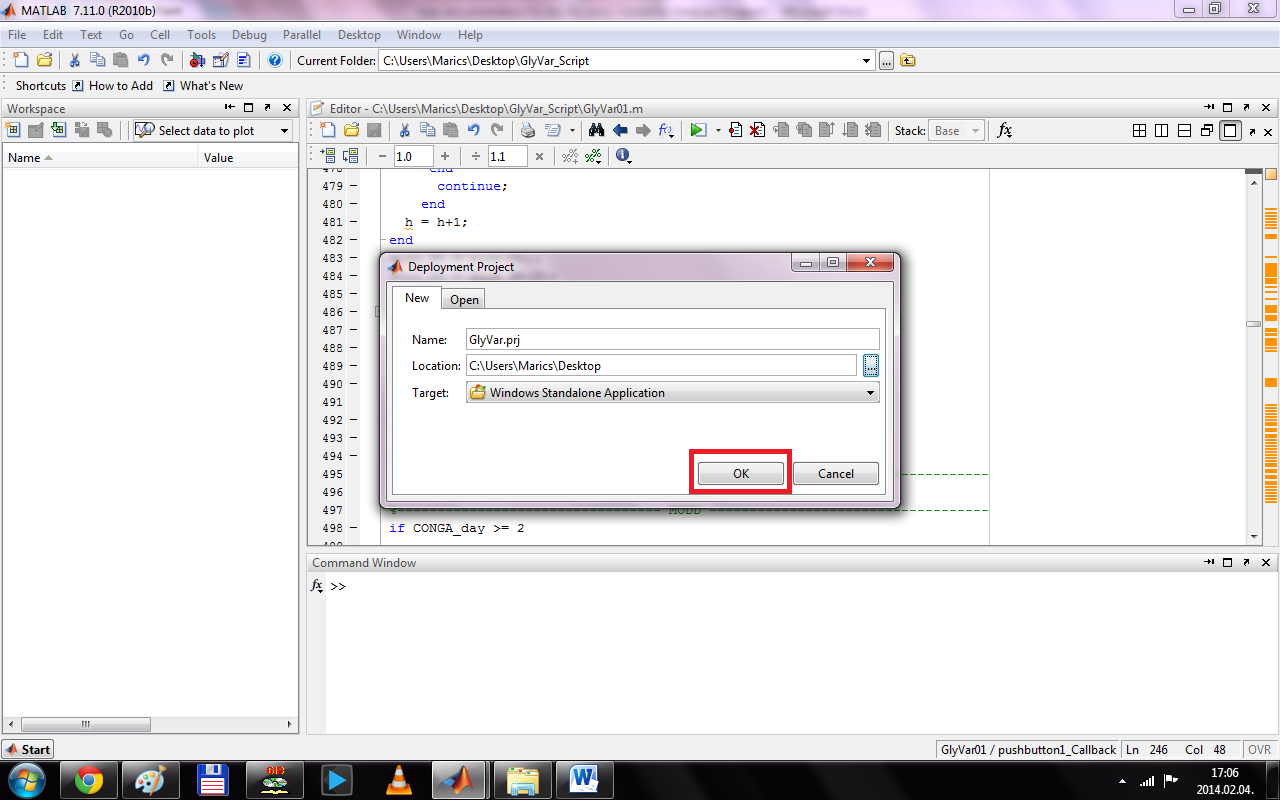


Step 2: Enter the project name and press OK


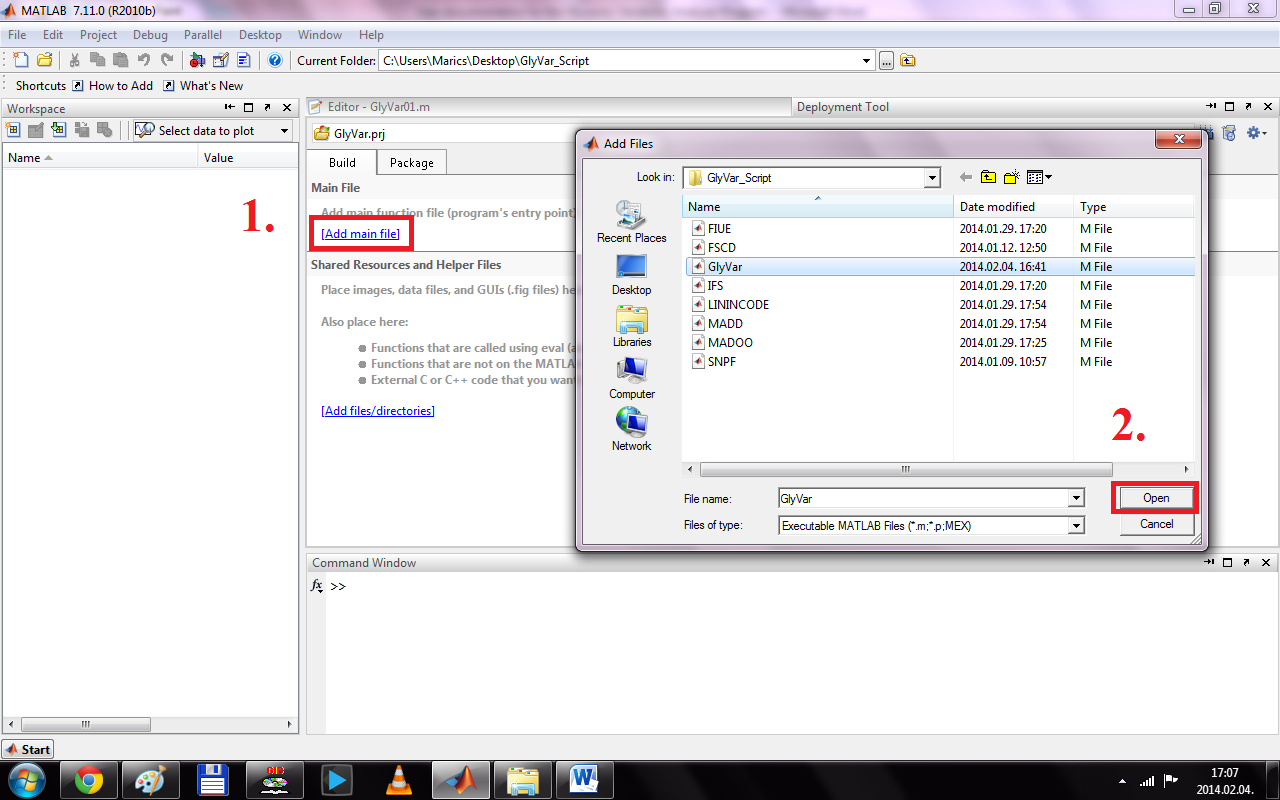


Step 3: Add the main file to the project.


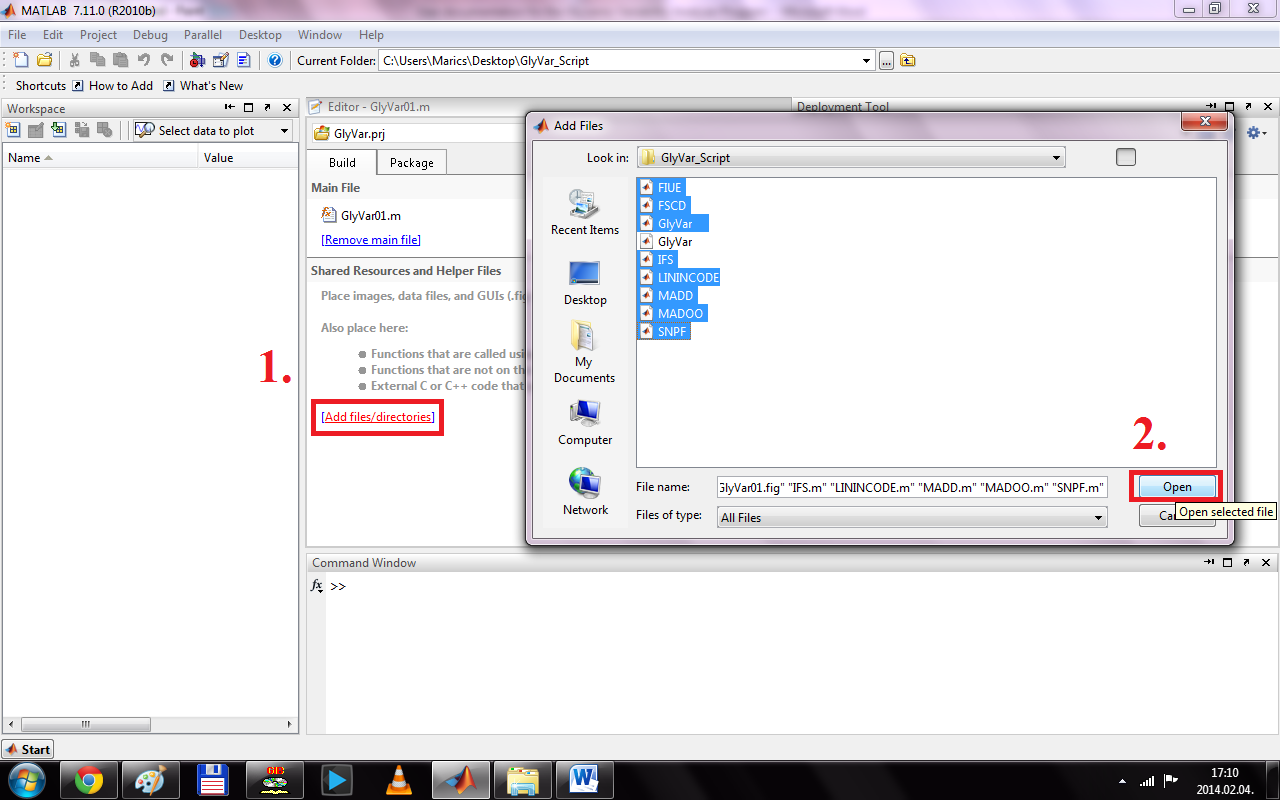


Step 4: Add the other necessary files to the project.


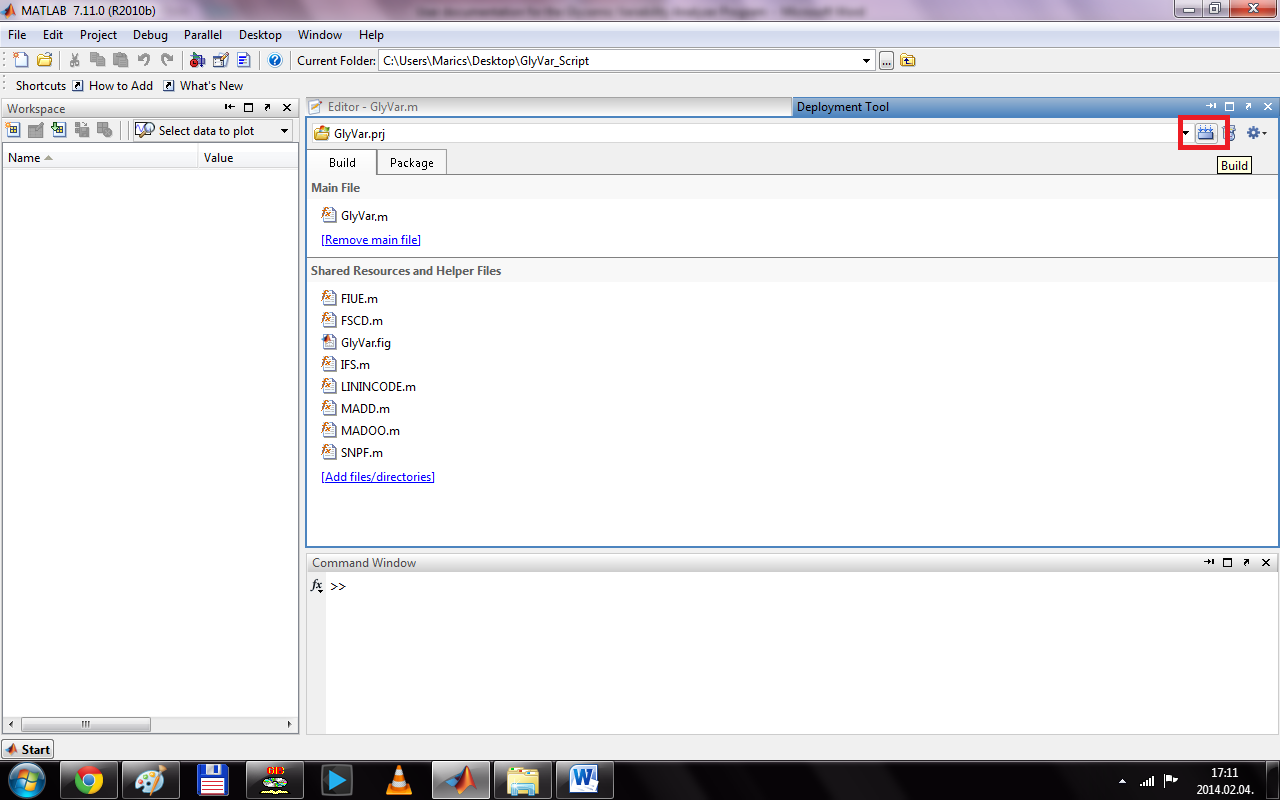


Step 5: Build the project (it can takes several minutes).


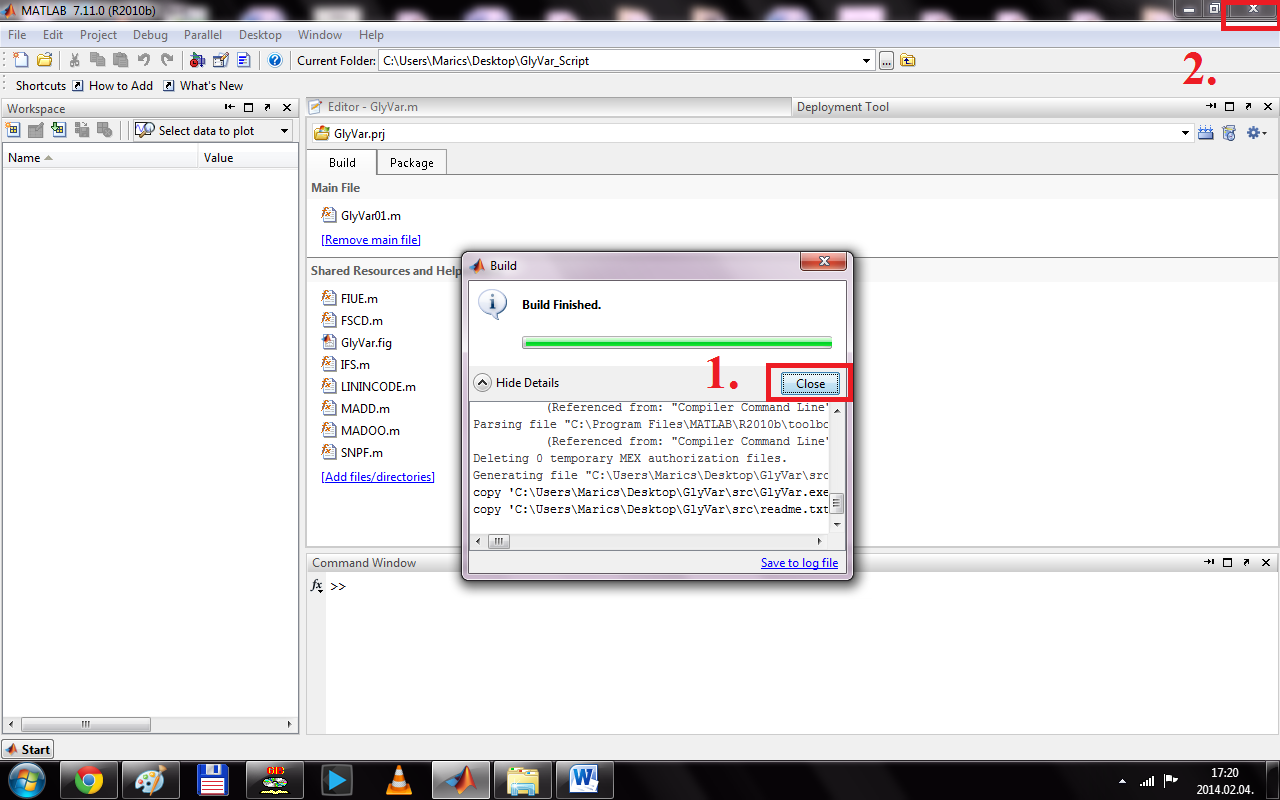


Step 6: After the building completion one can exit from the MATLAB.


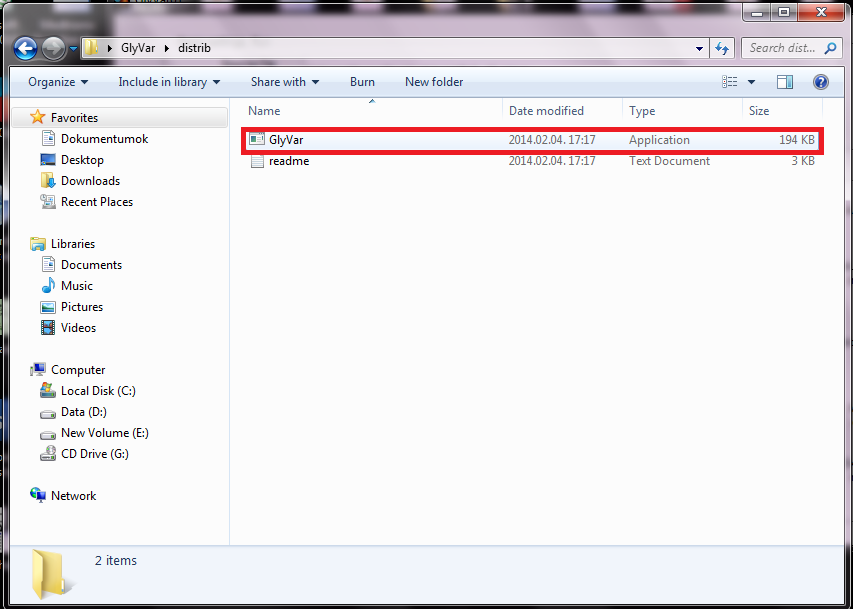


Step 7: Run the Windows Application. One can assign different icons to the application (eg.: CGMVariab.ico).
